# Rice Phytochrome B (OsPhyB) Negatively Regulates Dark- and Starvation-Induced Leaf Senescence

**DOI:** 10.3390/plants4030644

**Published:** 2015-09-01

**Authors:** Weilan Piao, Eun-Young Kim, Su-Hyun Han, Yasuhito Sakuraba, Nam-Chon Paek

**Affiliations:** Department of Plant Science, Plant Genomics and Breeding Institute, Research Institute of Agriculture and Life Sciences, Seoul National University, Seoul 151-921, Korea; E-Mails: vivianpanda622@gmail.com (W.P.); fndodpdy@snu.ac.kr (E.-Y.K.); bebe86@snu.ac.kr (S.-H.H.)

**Keywords:** rice, leaf senescence, phytochrome B, dark-induced senescence, starvation-induced senescence

## Abstract

Light regulates leaf senescence and light deprivation causes large-scale transcriptional reprogramming to dismantle cellular components and remobilize nutrients to sink organs, such as seeds and storage tissue. We recently reported that in Arabidopsis (*Arabidopsis thaliana*), Phytochrome-Interacting Factor4 (PIF4) and PIF5 promote dark-induced senescence and natural senescence by directly activating the expression of typical senescence-associated genes (SAGs), including *ORESARA1* (*ORE1*) and *ETHYLENE INSENSITIVE3* (*EIN3*). In contrast, phytochrome B (PhyB) inhibits leaf senescence by repressing PIF4 and PIF5 at the post-translational level. Although we found how red light signaling represses leaf senescence in Arabidopsis, it remains unknown whether PhyB and/or PhyA are involved in leaf senescence in rice (*Oryza sativa*). Here we show that rice *phyB* knockout mutants (*osphyB-1*, *-2*, and *-3*) exhibited an early senescence phenotype during dark-induced senescence, but an *osphyA* knockout mutant (*osphyA-3*) senesced normally. The RT-qPCR analysis revealed that several senescence-associated genes, including *OsORE1* and *OsEIN3*, were significantly up-regulated in *osphyB-2* mutants, indicating that OsPhyB also inhibits leaf senescence, like Arabidopsis PhyB. We also found that leaf segments of *osphyB-2* senesced faster even under light conditions. Supplementation with nitrogen compounds, such as KNO_3_ and NH_4_NO_3_, rescued the early senescence phenotype of *osphyB-2*, indicating that starvation is one of the major signaling factors in the *OsPhyB*-dependent leaf senescence pathway.

## 1. Introduction

In nature, the quality and quantity of sunlight vary depending on diurnal, seasonal, and local conditions. Thus, plants have evolved to use diverse sets of photoreceptors to perceive light of different wavelengths, enabling plants to fine-tune their growth and development over a large range of light conditions. Phytochromes (Phys), the photoreceptors that regulate the expression of large number of genes in a red/far-red (R/FR) light-dependent fashion, play a vital role in ensuring plants’ optimal acclimation to rapidly changing light conditions [1,2]. Phys are dimeric chromoproteins that exist in two different, stable conformations, depending on the wavelength of the incoming light [3]. The inactive Pr conformation can absorb red light and the active Pfr conformation can absorb far-red light [2]. The inactive Pr form localizes in the cytosol and the active Pfr form translocates into the nucleus to activate or repress its target genes [4], acting together with specific transcription factors (TFs) known as Phytochrome-Interacting Factors (PIFs; [5]).

The Arabidopsis (*Arabidopsis thaliana*) genome encodes five members of the Phy family (PhyA-PhyE); these proteins have a conserved *N*-terminal domain that functions in light perception and light signal transduction, and a *C*-terminal domain that is essential for dimerization and interaction with other proteins that function downstream of light signal transduction [4,6,7]. Among the five Phys, PhyA and PhyB regulate a wide range of light-dependent processes, such as seed germination, de-etiolation, hypocotyl elongation, shade avoidance, and flowering time [8].

In contrast to Arabidopsis, rice (*Oryza sativa*) has only three phys: OsPhyA, OsPhyB, and OsPhyC [9]. Knockout (KO) mutants of each rice Phy have been used to elucidate the physiological role of individual Phys [10,11]. The role of Phys in de-etiolation of Arabidopsis seedlings has been widely studied [12,13]. Similarly, OsPhyA and OsPhyB have important roles in de-etiolation of rice seedlings; thus, greening was strongly inhibited in *osphyA osphyB* double mutant seedlings under continuous red or far-red light [11]. OsPhys also contribute to rice architecture during development; for example, OsPhyA and OsPhyB regulate coleoptile elongation of young seedlings. The coleoptiles of *osphyB* mutant seedlings were significantly longer than those of wild-type (WT) seedlings under continuous red light conditions [11], and OsPhyB acts as a negative regulator in the brassinosteroid-regulated coleoptile elongation pathway [10]. In contrast, the coleoptiles of *osphyA* mutants were significantly longer than WT coleoptiles under far-red light conditions [11]. This difference between OsPhyA and OsPhyB is probably caused by the light specificity of each phytochrome and indicates that OsPhyA has a predominant role in perceiving FR light [14].

The *osphy* mutations also affect the angle of the leaf blade, a significant agronomic trait that alters planting density. The 2nd leaves in *osphyB* seedlings had a higher declination angle than the WT leaves. Although the *osphyA* seedlings had nearly the same angle as WT, the *osphyA osphyB* seedlings had a significantly greater declination angle than the *osphyB* seedlings [11], indicating that both OsPhyB and OsPhyA have important roles in leaf blade declination.

The OsPhys also affect floral induction; *osphyB* mutants flowered much earlier in both long-day (LD) and short-day (SD) conditions [11], like *phyB* mutants of other species, including Arabidopsis [15] and sorghum [16]. The *osphyC* null mutants also exhibited an early flowering phenotype under LD conditions, but flowered at approximately the same time as WT under SD conditions [11], indicating that OsPhyC has a photoperiod-dependent role in flowering time. In contrast with *osphyB* mutants, the *osphyA* mutants flowered at almost the same as WT under both LD and SD conditions. Notably, *osphyA osphyB* and *osphyA osphyC* double mutants flowered much earlier than *osphyB* and *osphyC* single mutants under LD conditions, respectively [11], indicating that OsPhyA contributes to OsPhyB and OsPhyC functions to suppress flowering in response to LD conditions.

Recently, we showed that Arabidopsis PhyB negatively regulates leaf senescence; *phyB*-KO mutants showed an early leaf senescence phenotype, while *PhyB*-overexpressing (*PhyB*-OX) plants showed delayed leaf yellowing during dark-induced senescence (DIS). However, KO mutants and OX plants of Arabidopsis *PhyA* did not show any senescence phenotype [17]. We also revealed that both PIF4 and PIF5 function as the central activators of DIS. It is well known that PhyB represses these two PIFs at the post-translational level during senescence [17]. PIF4 and PIF5 directly activate the expression of typical senescence-associated genes (SAGs), including *ORESARA1* (*ORE1*; [18]), *ETHYLENE INSENSITIVE3* (*EIN3*; [19]), and genes encoding abscisic acid (ABA)-responsive bZIP TFs, such as ABA INSENSITIVE5 (ABI5) and ENHANCED EM LEVEL (EEL; [20]). Furthermore, EIN3, ABI5, and EEL also directly activate *ORE1* expression, forming multiple coherent feed-forward loops for the activation of leaf senescence in Arabidopsis [17]. At almost the same time, Song *et al.* (2014) reported that, in addition to PIF4 and PIF5, PIF3 is also involved in the promotion of leaf senescence [21], but the PIF3 regulatory cascade is not clear yet.

Here we show that the *osphyB-2* mutants also exhibited an early senescence phenotype during DIS, similar to the Arabidopsis *phyB* mutants. Expression analysis revealed that rice homologs of *ORE1*, *EIN3*, *ABI5*, and *EEL* were significantly up-regulated in *osphyB-2* mutants. We also found that detached leaf segments of *osphyB-2* also showed an early senescence phenotype during light incubation. Furthermore, the early senescence phenotype of *osphyB-2* was partially recovered by supplementation with nitrogen compounds, indicating that at least in part, starvation-responsive signaling controls the *OsPhyB*-dependent induction of senescence. We discussed the possible mechanism of OsPhyB-regulating senescence in rice leaves.

## 2. Results

### 2.1. Leaf Blades of osphyB-2 Mutants Senesced Early during DIS

We previously reported that in Arabidopsis, *phyB* mutants senesced early and *phyB*-OX plants showed delayed leaf senescence during DIS [17], indicating that PhyB negatively regulates leaf senescence in Arabidopsis. Thus, OsPhyB likely also participates in the regulation of leaf senescence. To examine this, we obtained a T-DNA insertion mutant line (PFG_4A-02226.R) in which a single T-DNA fragment is inserted in the 3rd intron of *OsPhyB* (Supplemental Figure 1A), and the mutant leaves do not accumulate *OsPhyB* mRNA (Supplemental Figure 1B), indicating that this mutant (previously named *osphyB-2*) is an *OsphyB*-KO allele [10].

**Figure 1:**
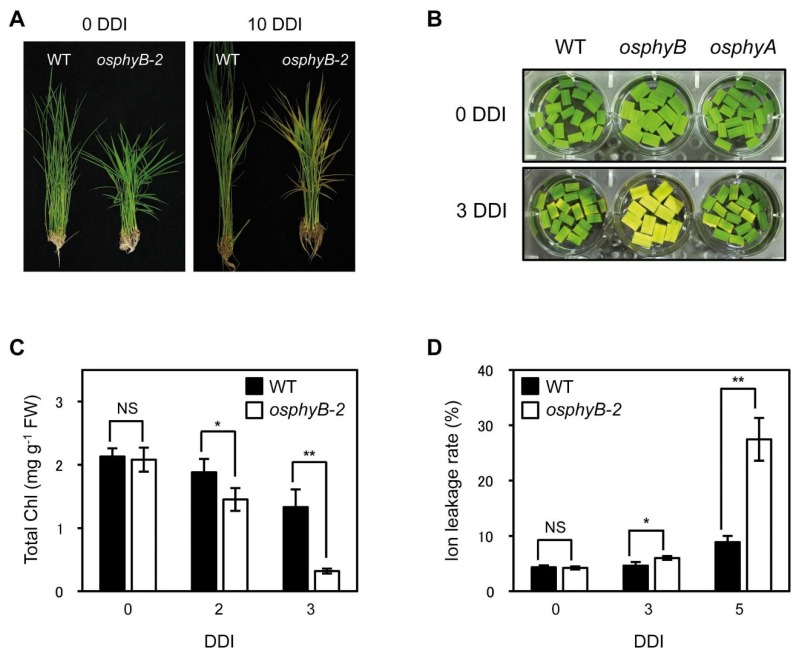
The *osphyB*-2 mutant exhibited an early senescence phenotype during DIS. (**A**) WT and *osphyB-2* whole plants were grown for 1 month under LD (14-h light/day) conditions and then were transferred to darkness at 28 °C for 10 days (10 DDI); (**B**) The color change that occurs in leaf segments of WT, *osphyB-2*, and *osphyA-3* during DIS; (**C**,**D**) The changes of total Chl level (**C**) and ion leakage rate (**D**) in the leaf segments of WT and *osphyB-2* during DIS. They were incubated on 3 mM MES (pH 5.8) buffer with the abaxial side up at 28 °C in darkness. Mean and SD values in (**C**,**D**) were obtained from at least five biological replicates. Asterisks indicate significant difference between WT and *osphyB-2* (Student’s *t*-test *p* values, *p < 0.05; **p < 0.01). NS, not significant; DDI, day(s) of dark incubation.

To compare this allele with other previously described *osphyB* mutants [11,22,23], we first measured the phenotypic characteristics of *osphyB-2* mutants, such as leaf angle, plant height, heading date, and seed fertility. The one-month-old *osphyB-2* mutants were shorter and had much wider leaf angles than the WT plants (Figure 1A, left panel; Supplemental Figure 2A,C), similar to other *osphyB* alleles [11]. For heading date and seed fertility of *osphyB-2* in the paddy field, we found that the *osphyB-2* mutants flowered much earlier (Supplemental Figure 3A), and had lower seed fertility than WT (Supplemental Figure 3B). Collectively, the phenotypic traits of *osphyB-2* are consistent with the phenotypes of other *osphyB* alleles [11,23].

Next, we examined the phenotype of *osphyB-2* mutants during DIS. The 4-week-old WT and *osphyB-2* whole plants were transferred to complete darkness. After 10 days of dark incubation (10 DDI), many leaves of *osphyB-2* became yellow, while most WT leaves retained their green color (Figure 1C). This early senescence phenotype of *osphyB-2* mutant was also examined using detached leaf segments. Leaf segments from one-month-old WT and *osphyB-2* were floated on MES buffer (pH 5.8), and incubated in darkness. The leaf segments of *osphyB-2* yellowed much faster than those of WT, showing a striking difference at 4 DDI (Figure 1B), similar to whole plants. Consistent with the visible phenotype, *osphyB-2* mutants had lower chlorophyll (Chl) levels (Figure 1C) and higher ion leakage rate, an indicator of membrane disintegration during senescence (Figure 1D). We also examined whether a different allele of *osphyB* showed the same early senescence phenotype, using the previously characterized allele *osphyB-1* [10]. We found that *osphyB-1* leaf segments also showed an early senescence phenotype during DIS (Supplemental Figure 4). These results indicate that OsPhyB is also involved in the repression of leaf senescence, like Arabidopsis PhyB [17].

We also obtained an *osphyA* mutant that has a single T-DNA fragment inserted in the 1st intron of *OsPhyA* (Supplemental Figure 5A; termed *osphyA-3*). This mutant did not accumulate *OsPhyA* transcripts in the leaves (Supplemental Figure 5B), indicating that it has *a* KO allele of *OsPhyA*. Unlike *osphyB-2*, however, *osphyA-3* mutants did not exhibit an altered senescence phenotype during DIS (Figure 1B), similar to the Arabidopsis *phyA* mutant [17].

### 2.2. Altered Gene Expression in osphyB-2 Mutants during DIS

We previously reported that Arabidopsis PhyB represses PIF4 and PIF5 at the post-translational level to delay leaf senescence in the light. In the dark, however, PhyB becomes inactive and the PIFs directly activate several key SAGs to induce leaf senescence cascade [17]. Thus, it is also possible that the expression levels of the key SAGs are altered in *osphyB-2* mutants during DIS.

**Figure 2:**
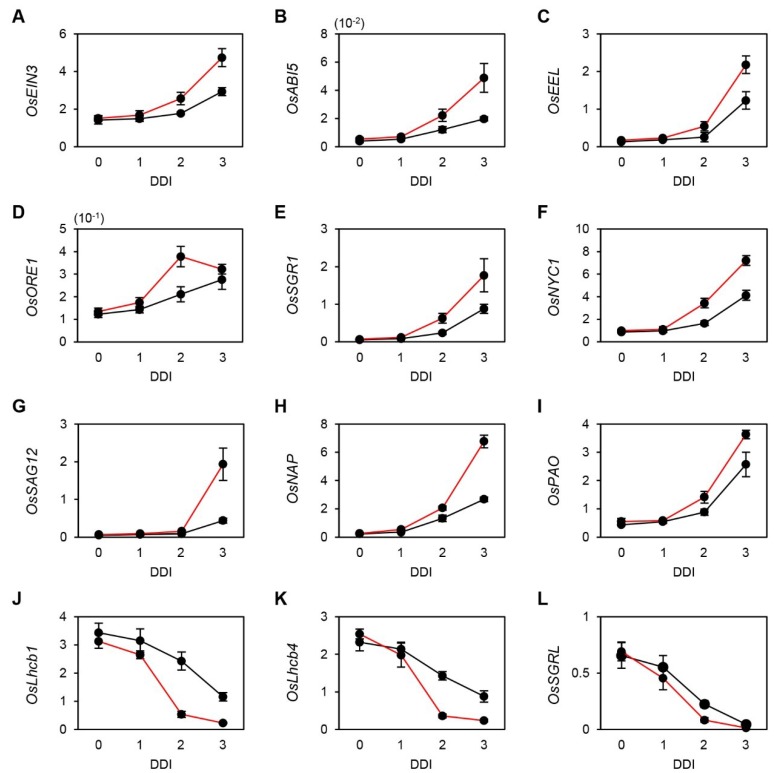
Expression of senescence-associated genes (SAGs) in WT and *osphyB-2* during DIS. Detached leaf segments from one-month-old WT (**black line**) and *osphyB-2* (**red line**) plants grown under LD conditions were incubated on 3 mM MES (pH 5.8) buffer with the abaxial side up at 28 °C in darkness, and were sampled at 0, 1, 2, and 3 DDI for RT-qPCR. RT-qPCR analysis was used to measure the relative transcript levels of *OsEIN3* (**A**), *OsABI5* (**B**); *OsEEL* (**C**); *OsORE1* (**D**); *OsSGR1* (**E**); *OsNYC1* (**F**); *OsSAG12* (**G**); *OsNAP* (**H**); *OsPAO* (**I**); *OsLhcb1* (**J**); *OsLhcb4* (**K**) and *OsSGRL* (**L**) and transcript levels were normalized to the transcript levels of *OsUBQ5*. Mean and SD values were obtained from more than three biological replicates. These experiments were repeated twice with similar results. DDI, day(s) of dark incubation.

To examine this possibility, we used reverse transcription-quantitative PCR (RT-qPCR) to measure the transcript levels of the key SAGs. In Arabidopsis, PIF4 and PIF5 directly activate *ABI5*, *EEL*, *EIN3*, and *ORE1*, and indirectly activate two Chl degradation-related genes, *STAY-GREEN1* (*SGR1*; [24]) and *NON-YELLOW COLORING1* (*NYC1*; [25]), during senescence [17]. Thus, we first checked the rice homologs of these six SAGs [26,27,28]. Before dark incubation (0 DDI), *OsEIN3*, *OsABI5*, *OsEEL*, *OsORE1*, *OsSGR1*, and *OsNYC1* showed similar expression levels in *osphyB-2* and WT plants. During dark incubation, however, the transcript levels of all six genes increased much faster in *osphyB-2* mutants than in WT (Figure 2A–F), indicating that OsPhyB regulates the PIF-dependent leaf senescence cascade, similar to the Arabidopsis PhyB-PIF regulatory module. We also checked the expression levels of other typical SAGs, *OsSAG12* [27], *OsNAP* [29], and *OsPAO* [30]. We found that these three genes were also significantly up-regulated in *osphyB-2* mutants during DIS (Figure 2G–I). In contrast, expression levels of three senescence down-regulated genes (SDGs), *OsLhcb1*, *OsLhcb4*, and *OsSGRL* [31] decreased much faster in *osphyB-2* mutants than in WT (Figure 2J–L). These results indicate that OsPhyB negatively regulates leaf senescence in the light by indirectly altering several SAGs and SDGs, similar to the Arabidopsis PhyB.

### 2.3. osphyB-2 Mutants Exhibited an Early Senescence Phenotype in the Light

**Figure 3:**
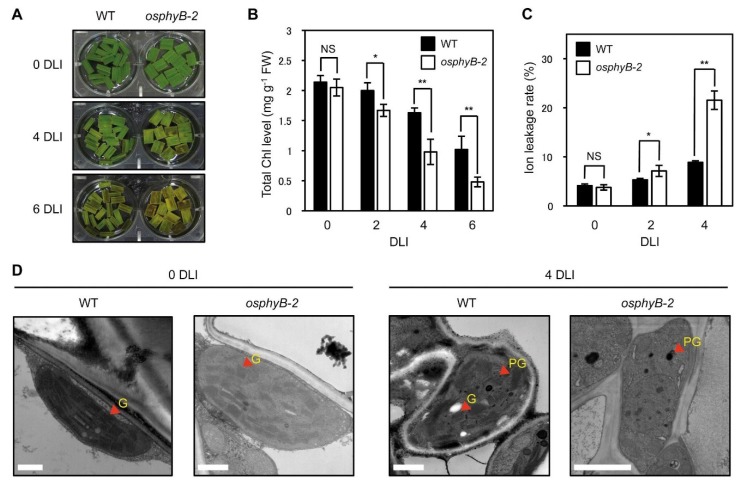
The *osphyB*-2 mutants senesced early even in light conditions. The changes of phenotype (**A**); total Chl level (**B**); ion leakage rate (**C**); and chloroplast structure (observed by transmission electron microscopy) (**D**) in WT and *osphyB-2* during starvation-induced senescence. Detached leaf segments from one-month-old WT and *osphyB-2* grown under LD conditions were incubated on 3 mM MES (pH 5.8) buffer with the abaxial side up at 28 °C under continuous light conditions, and were sampled at 0, 2, 4, and/or 6 DT for each experiment. (**B**,**C**) Mean and SD values were obtained from more than five biological replicates. Asterisks indicate significant difference between WT and *osphyB-2* (Student’s *t*-test *p* values, * *p* < 0.05; ** *p* < 0.01). (**D**) G, grana thylakoid; PG, plastoglobule. Scale bars = 1 μm. NS, not significant; DLI, day(s) of light incubation.

During DIS, detached leaves senesce much faster than attached leaves on whole plants [17,32], because the detached leaves are more susceptible to starvation. Thus, in addition to examining DIS, premature senescence is sometimes assessed in detached leaves incubated in the light [33]. Thus, we examined the senescence phenotype of *osphyB-2* in light conditions. Leaf segments from one-month-old WT and *osphyB-2* plants were floated on MES buffer (pH 5.8) and incubated under 7 days of light incubation (7 DLI). During incubation, yellowing of *osphyB-2* leaf segments began much faster (Figure 3A), with drastic decreases of Chl (Figure 3B), similar to the phenotype during DIS (Figure 1). The ion leakage rate in *osphyB-2* leaf segments was also higher than that of WT (Figure 3C). We also compared chloroplast structure of the WT and *osphyB-2* leaves at 0 and 7 DT. Transmission electron microscopy revealed that the WT and *osphyB-2* leaves had very similar chloroplast structures before light incubation (0 DT). At 7 DT, however, grana thylakoid structure in chloroplasts was hardly detectable in the *osphyB-2* leaves, but still remained intact in the WT leaves (Figure 3D). Together, these results indicate that OsPhyB is also involved in the regulation of starvation-induced leaf senescence under light conditions.

### 2.4. The Early Senescence Phenotype of osphyB-2 Mutants Was Recovered by Supplementation with Nitrogen Compounds

**Figure 4:**
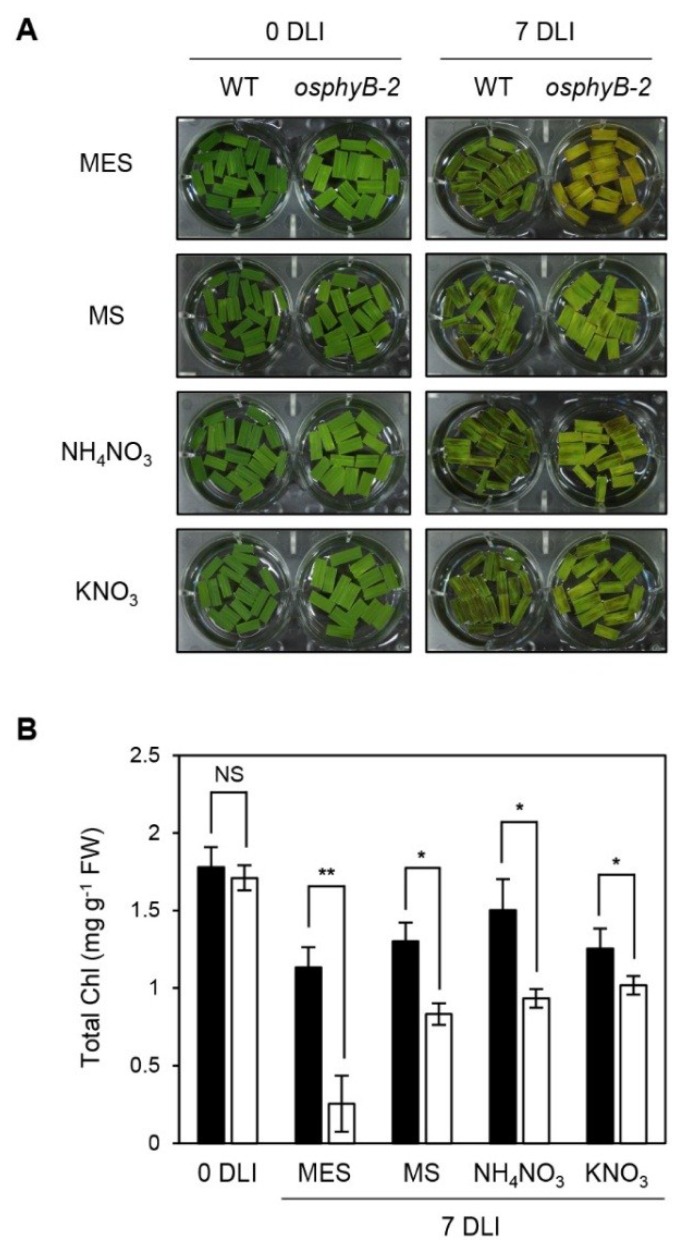
Early senescence phenotype of *osphyB*-2 was rescued by supplementation with nitrogen compounds. Detached leaf segments from one-month-old WT and *osphyB-2* plants grown under LD conditions were transferred to the 3 mM MES (pH 5.8) buffer only or supplemented with MS (2.3 g/L), NH_4_KO_3_ (1.65 g/L), or KNO_3_ (1.9 g/L), and incubated with the abaxial side up at 28 °C under continuous light for 7 days. The changes of phenotype (**A**) and total Chl level (**B**) in the leaf segments of WT and *osphyB-2* in each condition was observed and compared with the MES control; (**B**) Black and white bars indicate WT and *osphyB-2*, respectively. Mean and SD values were obtained from more than five biological replicates. Asterisks indicate significant difference between WT and *osphyB-2* (Student’s *t*-test *p* values, * *p* < 0.05; ** *p* < 0.01). NS, not significant; DLI, day(s) of light incubation.

Starvation appears to be closely related to the early senescence phenotype of *osphyB-2* mutants (Figure 3). To examine this possibility, we looked at the senescence phenotype of detached leaf segments of WT and *osphyB-2* plants in 3 mM MES buffer supplemented with Murashige-Skoog (MS) medium (2.3 g/L), a mixture of nutrients that are essential for plant growth [34]. As we expected, incubation in MS medium rescued the early senescence phenotype and low Chl levels of *osphyB-2* leaves in the light (Figure 4A,B).

MS medium contains a variety of essential nutrients for plant tissue culture, including the macronutrients nitrogen (NH_4_NO_3_ and KNO_3_), calcium (CaCl_2_), magnesium (MgSO_4_), and phosphorous (KH_2_PO_4_), and the micronutrients boron (H_3_BO_3_), cobalt (CoCl), copper (CuSO_4_), iron (FeSO_4_), manganese (MnSO_4_), iodine (KI), molybdenum (Na_2_MoO_4_), and zinc (ZnSO_4_), as well as the vitamins and organics i-inositol, niacin, pyridoxine, glycine, and thiamine [34]. To determine which component of MS medium causes the recovery of the early senescence phenotype of *osphyB-2*, we checked the senescence phenotype of *osphyB-2* leaves in 3 mM MES buffer containing each component under light conditions. We found that, among these components, the early senescence phenotype of *osphyB-2* was considerably recovered by supplementation with the two nitrogen compounds, KNO_3_ and NH_4_NO_3_ (Figure 4A,B, Supplemental Figure 6), but the phenotype was not recovered by supplementation with other important nutrients, such as i-Inositol or glycine [34]. In addition, we also confirmed the recovery of the early senescence phenotype of *osphyB-2* by the supplementation with KNO_3_ during DIS (Supplemental Figure 7), indicating that the signaling of nitrogen nutritional status has an important role in the regulation of *OsPhyB*-dependent leaf senescence.

**Figure 5:**
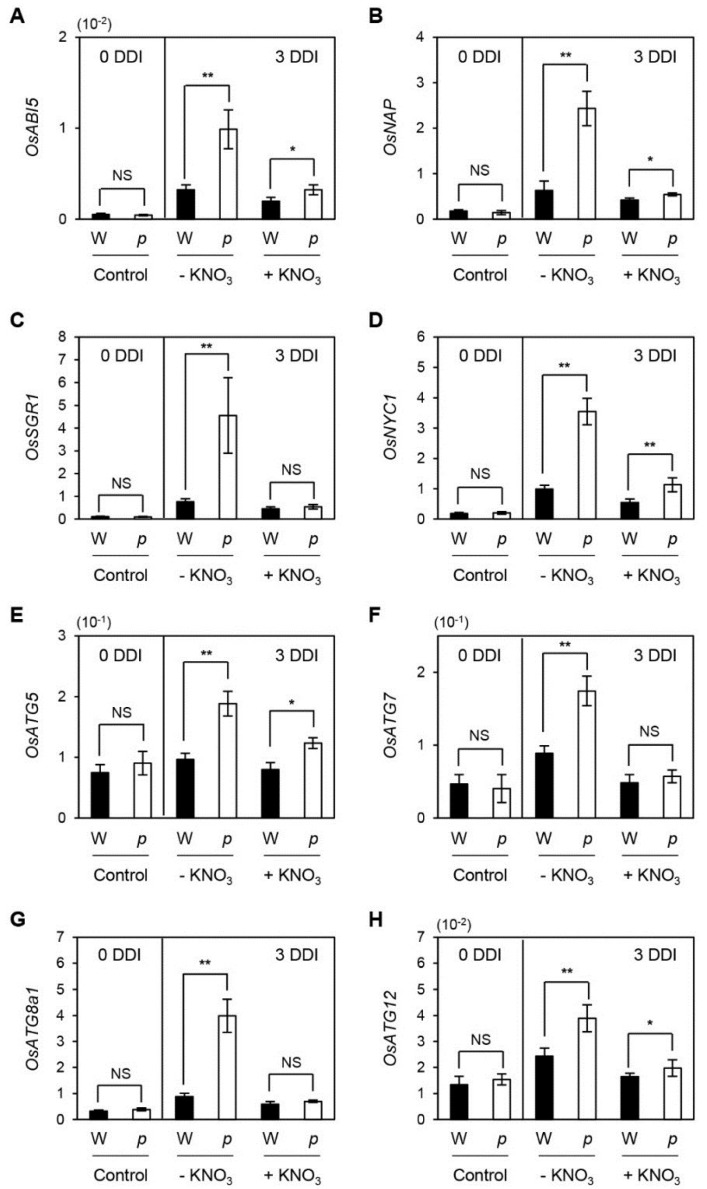
Up-regulation of SAGs in *osphyB-2* mutants during DIS rescued by supplementation with KNO_3_. Detached leaf segments from one-month-old WT (W; black bars) and *osphyB-2* (*p*; white bars) plants grown under LD conditions were floated on the 3 mM MES (pH 5.8) buffer without (−KNO_3_) or with 1.9 g/L KNO_3_ (+KNO_3_), with the abaxial side up at 28 °C in darkness, and were sampled at 0 (control) and 3 DDI for RT-qPCR. RT-qPCR analysis was used to measure the relative transcript levels of *OsABI5* (**A**); *OsNAP* (**B**); *OsSGR1* (**C**); *OsNYC1* (**D**); *OsATG5* (**E**); *OsATG7* (**F**); *OsATG8a1* (**G**); and *OsATG12* (**H**); and their transcript levels were normalized to the transcript levels of *OsUBQ5*. Mean and SD values were obtained from more than three biological replicates. These experiments were repeated twice with similar results. Asterisks indicate significant difference between WT and *osphyB-2* (Student’s *t*-test *p* values, * *p* < 0.05; ** *p* < 0.01). DDI, day(s) of dark incubation; NS, not significant.

We subsequently examined whether the up-regulation of SAGs in *osphyB-2* leaves during senescence is recovered by supplementation with nitrogen compounds. Consistent with the results of Figure 2, four SAGs (*OsNAP*, *OsABI5*, *OsSGR1*, and *OsNYC1*) were significantly up-regulated in *osphyB-2* leaves during dark incubation, but this increase of SAG mRNA levels was suppressed by supplementation with KNO_3_ (Figure 5A–D). We also checked the transcript levels of genes associated with autophagy, which is considered to function independently from the SGR-dependent Chl and chloroplast degradation pathways [35]. The expression levels of rice *AUTOPHAGY5* (*OsATG5*), *OsATG7*, *OsATG8a*, and *OsATG12* drastically increased in *osphyB-2* mutants during dark incubation without nitrogen supplementation (Figure 5E–H), indicating that OsPhyB regulates several key genes in the leaf senescence cascade, including Chl degradation and autophagy, and that nitrogen deficiency signaling partially overrules OsPhyB-dependent leaf senescence.

### 2.5. The Expression of OsPIF TFs during Senescence

Study of the PhyB-dependent leaf senescence pathway will require identification of downstream PIF components. We previously showed that in Arabidopsis, PhyB regulates PIF4 and PIF5 at the post-translational level [17]. Furthermore, Song *et al.* (2014) showed that Arabidopsis PIF3 is also involved in inducing leaf senescence [21]. Thus, KO mutants of each of the three PIFs showed a delayed senescence phenotype, while OX plants senesced early under both DIS and natural senescence conditions [17,21]. The expression levels of the three *PIFs* also significantly increased during DIS or natural senescence conditions [17,21]. However, Arabidopsis PIF1 is not involved in leaf senescence, because we found that *pif1*-KO mutants did not show any senescence phenotype during DIS [17]. We found by RT-qPCR analysis that the expression of *PIF1* is significantly down-regulated during DIS (Supplemental Figure 8). Thus, we found that examination of the expression levels of *PIF* genes during senescence is a useful approach for identification of senescence-associated *PIF* genes.

Previous phylogenetic analysis revealed that four rice PIFs, OsPIL11 (Os12g0610200), OsPIL12 (Os03g0639300), OsPIL13 (Os03g0782500), and OsPIL14 (Os07g143200), have high amino acid sequence similarity to Arabidopsis PIF4 and PIF5, and two rice PIFs, OsPIL15 (Os01g0286100) and OsPIL16 (Os05g0139100), have high similarity to Arabidopsis PIF3 [36]. Thus, these rice PIFs may be functional homologs of Arabidopsis PIF3, PIF4, and PIF5 (hereafter Arabidopsis senPIFs). Indeed, Arabidopsis transgenic plants overexpressing *OsPIL11*, *OsPIL12*, *OsPIL13*, *OsPIL14*, or *OsPIL15* exhibited a long hypocotyl phenotype [36], similar to *PIF3*-, *PIF4*-, and *PIF5*-OX plants [37,38].

**Figure 6:**
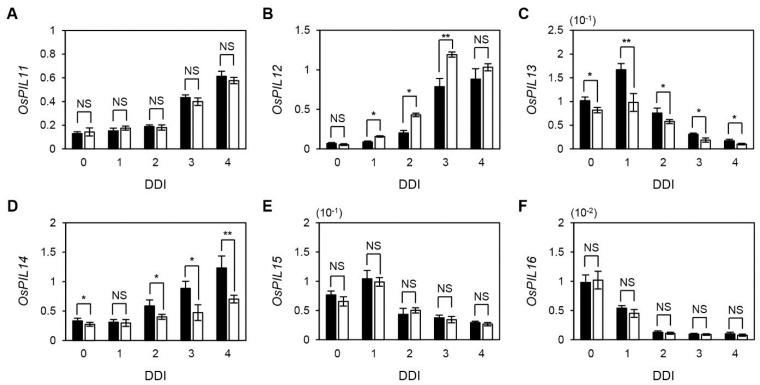
Expression of six rice *PIF* genes during DIS. Detached leaf segments from one-month-old WT and *osphyB-2* plants grown under LD conditions were incubated on the 3 mM MES (pH 5.8) buffer with the abaxial side up at 28 °C in darkness, and were sampled at 0 to 4 DDI for RT-qPCR. RT-qPCR was used to measure the relative transcript levels of *OsPIL11* (**A**); *OsPIL12* (**B**); *OsPIL13* (**C**); *OsPIL14* (**D**); *OsPIL15* (**E**); and *OsPIL16* (**F**); and transcript levels were normalized to the transcript levels of *OsUBQ5*. Black and white bars indicate WT and *osphyB-2*, respectively. Mean and SD values were obtained from more than three biological replicates. Asterisks indicate significant difference between WT and *osphyB-2* (Student’s *t*-test *p* values, * *p* < 0.05; ** *p* < 0.01). These experiments were repeated twice with similar results. DDI, day(s) of dark incubation; NS, not significant.

Thus, we first checked the expression of these six *OsPIF* genes during DIS. The transcript levels of *OsPIL11*, *OsPIL12*, and *OsPIL14* continuously increased until 4 DDI (Figure 6A,B,D). The expression peaks of *OsPIL13* and *OsPIL15* were at 1 DDI and their expression levels gradually decreased afterward (Figure 6C,E). In contrast, the expression levels of *OsPIL16* drastically decreased until 4 DDI (Figure 6F), similar to the expression pattern of Arabidopsis *PIF1* (Supplemental Figure 8). Interestingly, *OsPIL12* was up-regulated, while *OsPIL13* and *OsPIL14* were down-regulated in *osphyB-2* during DIS (Figure 6B–D), similar to Arabidopsis *PHYTOCHROME-INTERACTING FACTOR 3-LIKE 1* (*PIL1*), as reported previously [17].

We also checked the expression levels of these six *OsPIF* genes in senescent leaves. Consistent with the results under DIS conditions, *OsPIL11*, *OsPIL12*, *OsPIL13*, and *OsPIL14* were expressed at higher levels in senescent segments of leaves than that in non-senescent green segments of leaves (Supplemental Figure 9A–E). In contrast, the expression levels of *OsPIL15* and *OsPIL16* were higher in non-senescent segments (Supplemental Supplemental Figure 9F,G). Thus, we identified candidate senescence-associated *PIF* genes, such as *OsPIL11*, *OsPIL12*, *OsPIL13*, and *OsPIL14*, which are possibly functional homologs of Arabidopsis *PIF3*, *PIF4*, and *PIF5* in leaf senescence signaling.

## 3. Discussion

We previously found the Arabidopsis PhyB-PIFs regulatory module of leaf senescence. PIF proteins are stable in the dark but rapidly degraded upon exposure to light; the active form of Phy (Pfr) promotes the light-dependent destabilization of PIFs, via the ubiquitin-26S proteasome pathway [39,40]. During DIS in Arabidopsis, PhyB changes to the inactive form (Pr), which cannot move to the nucleus. As a result, senescence-associated PIFs (PIF3, PIF4, and PIF5) increase, leading to the activation of regulatory cascades that induce leaf senescence [17,21]. Furthermore, PIF levels increase more in *phyB* null mutants than in WT; therefore, Arabidopsis *phyB* mutants exhibited an early senescence phenotype during DIS [17]. In this study, we found that *osphyB-2* mutants senesced earlier during DIS (Figure 1), similar to the Arabidopsis *phyB* mutants [17], indicating that phyB-PIF also has an important role in the regulation of leaf senescence in rice.

In the Arabidopsis leaf senescence pathway regulated by PhyB-PIF, we previously identified several key downstream genes, including *ORE1*, *EIN3*, *ABI5*, *EEL*, *SGR1*, and *NYC1* [17]. Furthermore, EIN3 directly activates *NAP* and *ORE1* during leaf senescence [41]. In this study, we confirmed by RT-qPCR that rice homologs of these genes were significantly up-regulated during DIS (Figure 2), indicating that PhyB regulation of leaf senescence cascade is highly conserved in Arabidopsis and rice, although further examination of this pathway will require the identification of OsPhyB-regulated senPIFs. We found several key genes in PhyB-PIF-regulating leaf senescence cascade in both Arabidopsis and rice, but other SAGs may also participate in this regulatory network. In recent years, many SAGs (such as NAC and WRKY TFs, and genes associated with light signaling, Phy signaling, Chl degradation, and chloroplast maintenance) have been identified in Arabidopsis and other plant species [17,42,43,44]. Further identification of downstream genes of senPIFs will improve our understanding of the PhyB-PIF cascade regulating leaf senescence.

We found that the early senescence phenotype of *osphyB-2* also occurred during starvation-induced senescence under continuous light conditions (Figure 3). Furthermore, the early senescence phenotype of *osphyB-2* was rescued by supplementation of nitrogen compounds, such as KNO_3_ and NH_4_NO_3_, under both light and dark conditions (Figure 4; Supplemental Figure 7), indicating that nitrogen deficiency is one of the major signaling cascades in OsPhyB-dependent leaf senescence. Nitrogen is an essential macronutrient in plant growth and development and plants senesce earlier under low nitrogen conditions [39,40], because under nitrogen-limited conditions, plants have to save nitrogen for the development of storage tissues, such as seeds and sink organs. Conversely, supplying nitrogen can reverse the progress of senescence [45,46]. Balazadeh *et al.* (2014) reported that the levels of secondary metabolites (amino acids and sugars) and SAGs in the leaves of Arabidopsis changed dramatically under *N*-deficient conditions, but these changes were rapidly reversed following nitrogen supply [45]. In the case of *osphyB-2* leaves, we first speculated that they have lower innate nitrogen levels compared with WT. In *osphyB-2* leaves, however, the protein levels of RbcL, the largest nitrogen storage protein, which contains around 75% of the total nitrogen in plants [47], was almost the same as in WT leaves (Figure 3). Thus, nitrogen signaling has an important role in OsPhyB-dependent DIS and starvation-induced senescence in the light, although it is possible that other metabolites and/or transcriptional signaling pathways also affect these phenotypes.

In this study, we also examined the expression of six *OsPIF*s during DIS and in naturally senescing leaves to identify candidate senPIF genes in rice (Figure 6 and Supplemental Figure 9). Expression of *OsPIL13* showed the most similarity to expression of Arabidopsis senPIFs. Similar to *OsPIL13*, in Arabidopsis three senPIFs rapidly increased in early DIS and then gradually decreased [17,21], indicating that senPIFs have an important role in the early stages of DIS. The *OsPIL13* expression level is also controlled by circadian rhythm [36], similar to the expression of Arabidopsis *PIF4* and *PIF5* [48,49]. The expression patterns of *OsPIL11*, *OsPIL12*, and *OsPIL14* differed somewhat from those of *OsPIL13* and Arabidopsis senPIFs. The mRNA levels of these three *OsPILs* continuously increased until 4 DDI (Figure 6). However, their expression levels were all higher in senescent segments of leaves. Thus, it is possible that four OsPIFs (OsPIL11, OsPIL12, OsPIL13, and OsPIL14) are involved in the leaf senescence cascade, like Arabidopsis senPIFs. Interestingly, expression levels of *OsPIL12*, *OsPIL13*, and *OsPIL14* were differentially regulated in *osphyB-2* during DIS (Figure 6), similar to the expression of *PIF* genes in Arabidopsis. For example, PIF4 and PIF5 directly regulate *PIL1* expression [50]. Thus, *PIL1* mRNA levels were much lower in *pif1 pif3 pif4 pif5* quadruple mutants and much higher in *phyB* mutants, compared with WT, during DIS [17]. As in the case of Arabidopsis *PIL1*, it is possible that direct regulation by other OsPIFs causes the altered expression of *OsPIL12*, *OsPIL13*, and *OsPIL14* in *osphyB-2* mutants.

Isolation of KO mutants and OX plants of these *OsPIFs* will provide essential information to elucidate their physiological functions, but so far related reports remain very limited. One of these reports describes the development and phenotypic analysis of rice transgenic plants overexpressing *OsPIL13* ([51]; it was termed *OsPIL1* in this reference). *OsPIL13*-OX plants showed increased internode elongation, caused by differences in cell size. Microarray analysis revealed that expression of some cell wall-related genes, which are responsible for cell elongation, increased in *OsPIL13*-OX plants [51]. They also found that the expression of *OsPIL13* dramatically decreased under drought and cold stress conditions [51], indicating that OsPIL13 has an important role in cell elongation and abiotic stress responses. This function of OsPIL13 is similar to the functions of Arabidopsis PIF3, PIF4, and PIF5; their OX plants show a long-hypocotyl phenotype [37,38] and their knockout mutants show a short-hypocotyl phenotype [52]. Thus, OsPIL13 could act as a senescence-regulating TF, similar to Arabidopsis PIF3, PIF4, and PIF5 [17,21]. In addition to cell elongation and leaf senescence, Arabidopsis PIFs also regulate other biological events, including de-etiolation, flowering time, Phy responses, and stress responses [5,17,48,53,54,55]. Isolation and analyses of mutants and OX plants of *OsPIF* genes will help elucidate the multiple functions of OsPIFs in rice development.

## 4. Experimental Section

### 4.1. Plant Materials and Growth Conditions

The WT *japonica* rice cultivar “Dongjin” and *osphyB-2* mutants were grown in the growth chamber under LD conditions (14-h light/day) or in the paddy field under natural long days (>14 h light/day) in Suwon, South Korea (37° N latitude). The T-DNA insertion *osphyB-1* (PFG_2D-20484.R), *osphyB-2* (PFG_4A-02226.R) and *osphyA-3* (PFG_3A-16812.R) mutants were obtained from the Crop Biotech Institute at Kyung Hee University, Korea [56,57].

### 4.2. DIS and Starvation-Induced Senescence Treatments

For the DIS and starvation-induced senescence experiments, we used whole plants or detached leaf segments at an early, vegetative phase when the plants are one-month old, about two months before the heading date of *osphyB* mutants. For the DIS experiments using whole plants, 4-week-old WT and *osphyB-2* plants were incubated in complete darkness on 3 mM MES (pH 5.8) buffer. In addition, detached leaf segments from 1-month-old plants grown under LD conditions were incubated on 3 mM MES (pH 5.8) buffer with the abaxial side up at 28 °C in complete darkness for DIS or in the light for starvation-induced senescence. The 3 mM MES buffer (pH 5.8) containing MS (2.3 g/L), NH_4_KO_3_ (1.65 g/L), or KNO_3_ (1.9 g/L) were used for the recovery assay of DIS and starvation-induced senescence.

### 4.3. Chl Quantification

For the measurement of total Chl levels, pigments were extracted from leaf tissues with 80% ice-cold acetone. Chl concentrations were determined by spectrophotometry as described previously [58].

### 4.4. Measurement of Ion Leakage Rate

Ion leakage rate was measured as previously described [27]. Briefly, membrane leakage was determined by measurement of electrolytes (or ions) leaking from rice leaf segments (1 cm^2^). Three leaf segments from each treatment were immersed in 6 mL of 0.4 M mannitol at room temperature with gentle shaking for 3 h, and initial conductivity of the solution was measured with a conductivity meter (CON 6 METER, LaMotte Co., Chestertown, MD, USA). Total conductivity was determined after sample incubation at 85 °C for 20 min. The ion leakage rate was expressed as the percentage of initial conductivity divided by total conductivity.

### 4.5. Transmission Electron Microscopy

To perform transmission electron microscopy, a previously described method [59] was used with some modifications. Small leaf pieces were fixed with modified Karnovsky’s fixative (2% paraformaldehyde, 2% glutaraldehyde, and 50 mM sodium cacodylate buffer, pH 7.2). After this, samples were washed with 0.05 M sodium cacodylate buffer, pH 7.2 three times at 4 °C for 10 min. The samples were post-fixed at 4 °C for 2 h with 1% osmium tetroxide in 50 mM sodium cacodylate buffer, pH 7.2, and washed twice with distilled water at room temperature. Samples were stained *en bloc* in 0.5% uranyl acetate at 4 °C overnight and dehydrated in an ethanol gradient with propylene oxide, then infiltrated with Spurr’s resin. Samples were polymerized at 70 °C for 24 h and sectioned with an ultramicrotome (MT-X). The sections were mounted on copper grids, and stained with 2% uranyl acetate for 7 min and with Reynolds’ lead citrate for 7 min. Micrographs were made using a LIBRA 120 transmission electron microscope.

### 4.6. SDS-PAGE and Immunoblot Analysis

Protein extracts were prepared from leaf tissues. To extract total proteins, leaf tissues were ground in liquid nitrogen and 10 mg aliquots were homogenized with 100 μL of sample buffer (50 mM Tris pH 6.8, 2 mM EDTA, 10% glycerol, 2% SDS, and 6% 2-mercaptoethanol). Homogenates were centrifuged at 10,000× *g* for 3 min, and supernatants were denatured at 80 °C for 5 min. 4 μL of each sample was subjected to 12% (w/v) polyacrylamide SDS-PAGE and resolved proteins were electroblotted onto a Hybond-P membrane (GE Healthcare). Antibodies against the photosystem proteins Lhcb1 and Lhca1 (Agrisera, Vannas, Sweden) were used for immunoblot analysis. The level of each protein was examined using the ECL system with WESTSAVE (AbFRONTIER, Seoul, Korea) according to the manufacturer’s protocol.

### 4.7. Reverse Transcription and Quantitative Real-Time PCR (RT-qPCR) Analysis

Total RNA was extracted from 4-week-old plants with the MG Total RNA Extraction Kit (Macrogen, Korea). First-strand cDNAs were synthesized with 2 μg of total RNA in a 25 µL volume using M-MLV reverse transcriptase and oligo(dT)_15_ primer. The 20 µL of qPCR mixture contained 2 µL of the RT mixture, 10 µL of 2× Go Tag PCR mix (Roche) and 0.25 µL of the primers. The qPCR was performed on the Light Cycler 2.0 (Roche Diagnostics, Basel, Switzerland). The qPCR conditions were 95 °C for 2 min, followed by 45 cycles at 95 °C for 5 s, 59 °C for 15 s, and 72 °C for 10 s. RT-qPCR analysis was used to measure the relative transcript levels of genes examined in this study, and the transcript levels were normalized to the transcript levels of *OsUBQ5* (Os01g0328400). Primers used for RT-qPCR analysis are listed in Supplemental Table 1.

## Supplementary Files

Supplementary File 1

## Gene Information

Sequence data from this article can be found in the National Center for Biotechnology Information (NCBI): *OsABI5*, Os01g0859300; *OsATG5*, Os02g0117800; *OsATG7*, Os01g0614900; *OsATG8a1*, Os07g0512200; *OsATG12*, Os06g0205000; *OsEEL*, Os07g0686100; *OsEIN3*, Os03g0324200; *OsLhcb1*, Os01g0601600; *OsLhcb4*, Os07g0558400; *OsNAP*, Os03g0327800; *OsNYC1*, Os01g0227100; *OsORE1*, Os04g0460600; *OsPAO*, Os03g0146400; *OsSAG12*, Os04g0208200; *OsSGR*, Os09g0532000; *OsPhyA*, Os03g0719800; *OsPhyB*; *OsPIL11*, Os12g0610200; *OsPIL12*, Os03g0639300; *OsPIL13*, Os03g0782500; *OsPIL14*, Os07g143200; *OsPIL15*, Os01g0286100; *OsPIL16*, Os05g0139100; *OsSGRL*, Os04g0692600; *OsUBQ5*, Os01g0328400.
