# Supplementary material for: Rice Phytochrome B (OsPhyB) Negatively Regulates Dark- and Starvation-Induced Leaf Senescence
Source: Plants (Basel). 2015 Sep 1;4(3):644–63. doi: 10.3390/plants4030644 (PMC4844404; doi:10.3390/plants4030644)
Supplement: Supplementary File 1 [file plants-04-00644-s001.pdf]

Supplementary Information

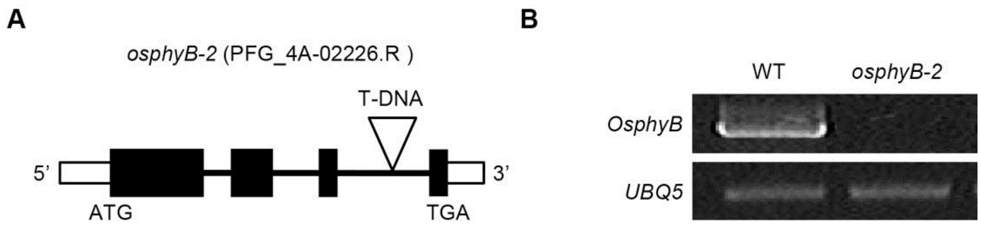

Supplemental Figure 1. Characterization of T-DNA insertion in *osphyB-2* allele. (**A**) Gene structure and T-DNA insertion site in the 3rd intron of Os*PhyB* (PFG_4A-02226.R); (**B**) The absence of *OsPhyB* transcripts in *osphyB-2* mutants was confirmed by RT-PCR. *UBQ5* was used as an internal control.

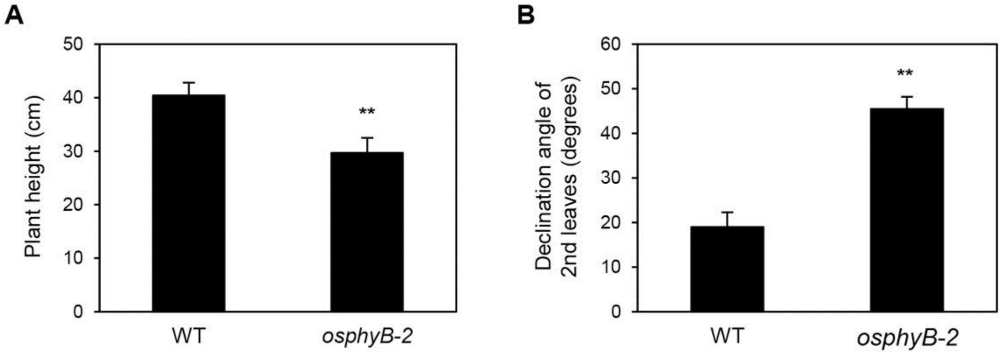

Supplemental Figure 2. Plant height and leaf angle of *osphyB-2* mutants. Plant height (**A**) and declination angle were examined for the 2nd leaves (**B**) of one-month-old WT (a parental japonica cultivar “Dongjin”) and *osphyB-2* mutants grown under LD (14-h light/day) conditions. Mean and SD values were obtained from more than five biological replicates. Asterisks indicate significant difference between WT and *osphyB-2* (Student’s *t*-test *p* values, ** $p < 0.01$).

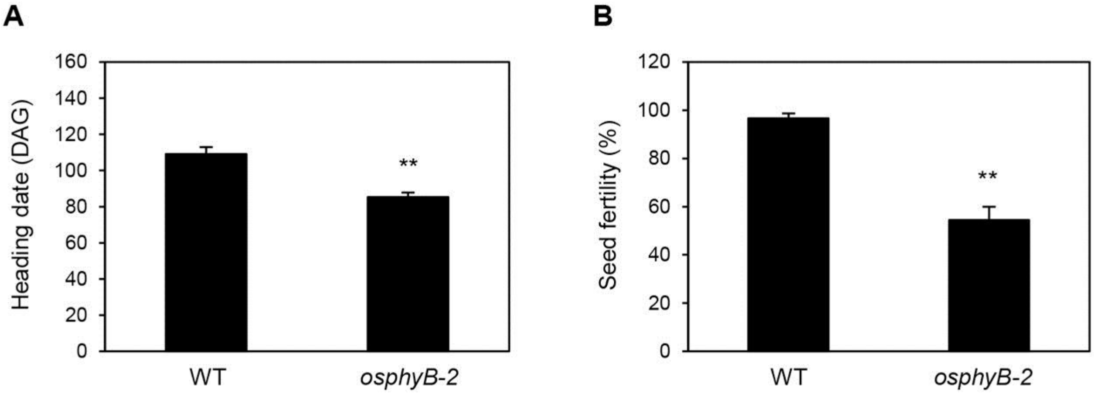

Supplemental Figure 3. Heading date and seed fertility of *osphyB-2* mutant.Heading date (**A**) and seed fertility (**B**) of WT (a *japonica* cultivar “Dongjin”) and *osphyB-2* was examined in natural paddy field conditions. Mean and SD values were obtained from more than five biological replicates. Asterisks indicate significant difference between WT and *osphyB-2* (Student’s *t*-test *p* values, ** $p < 0.01$). DAG, day(s) after germination.

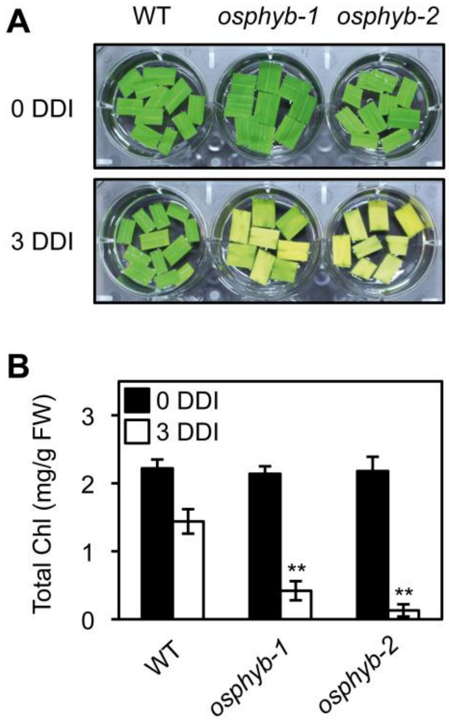

Supplemental Figure 4. The *osphyB-1* mutant exhibited an early senescence phenotype during DIS. (**A**,**B**) The changes of leaf color (**A**) and total Chl (**B**) in WT and *osphyB-1* leaf segments during DIS. The leaf segments were incubated on 3 mM MES (pH 5.8) buffer with the abaxial side up, at 28 ℃ in darkness. Mean and SD values were obtained from at least five biological replicates. Asterisks indicate significant difference between WT and *osphyB-1* mutant (Student's *t*-test *p* values, ***** $p < 0.05$; ****** $p < 0.01$). NS, not significant; DDI, day(s) of dark incubation.

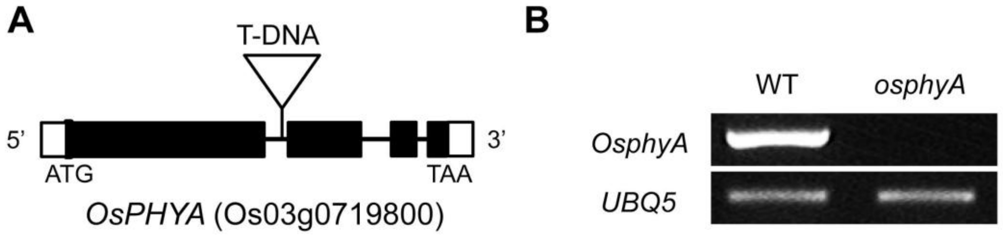

Supplemental Figure 5. The *osphyA-3* mutant is a knockout allele. (**A**) Gene structure and T-DNA insertion site in the 1st intron of *OsPhyA* (LOC_Os03g0719800); (**B**) The absence of *OsPhyA* transcripts in *osphyA-3* mutants was confirmed by RT-PCR. *UBQ5* was used as an internal control.

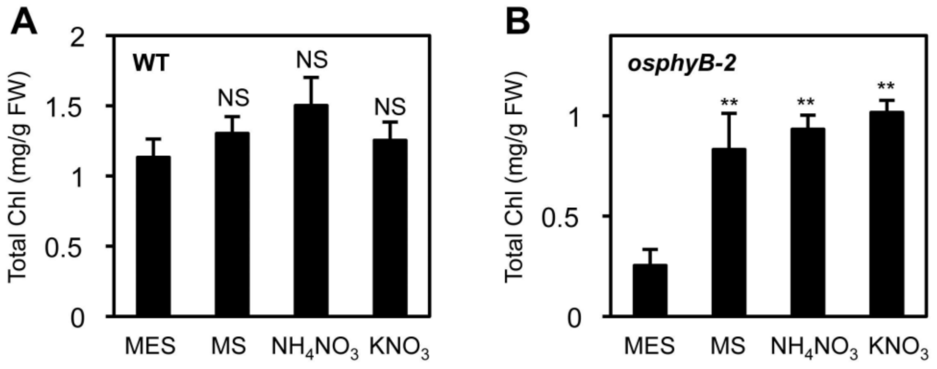

Supplemental Figure 6. The early senescence phenotype of *osphyB-2* was rescued by supplementation with nitrogen compounds. Detached leaf segments from one-month-old WT and *osphyB-2* plants grown under LD conditions were transferred to the 3 mM MES (pH 5.8) buffer only or supplemented with MS (2.3 g/L), NH_4KO_3 (1.65 g/L), or KNO_3 (1.9 g/L), and incubated with the abaxial side up at 28 ℃ under continuous light for 7 days. The changes of phenotype and total Chl levels in the leaf segments of WT (**A**) and *osphyB-2* (**B**) in each condition were observed and compared with the MES control. Mean and SD values were obtained from more than five biological replicates. Asterisks indicate significant difference between WT and *osphyB-2* (Student's *t*-test *p* values, ** $p < 0.01$). NS, not significant.

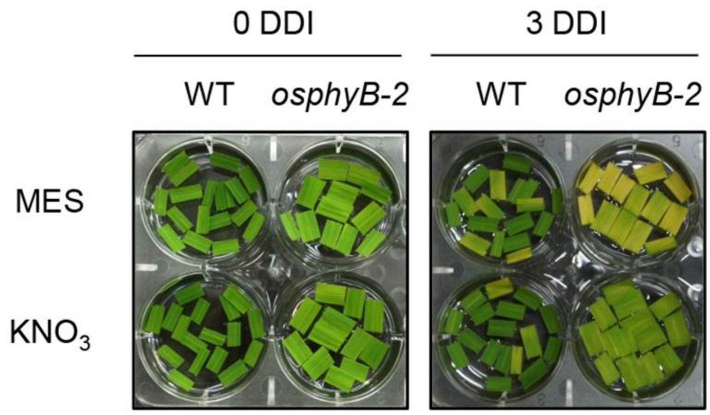

Supplemental Figure 7. The effect of nitrogen supplements on the leaf phenotype of *osphyB-2* during DIS. Leaf segments from 1-month-old WT and *osphyB-2* grown under LD conditions were floated on 3 mM MES (pH 5.8) buffer only (control) or supplemented with 1.9 g/L KNO_3 (+KNO_3), with the abaxial side up at 28 ℃ in darkness for 3 days. DDI, day(s) of dark incubation.

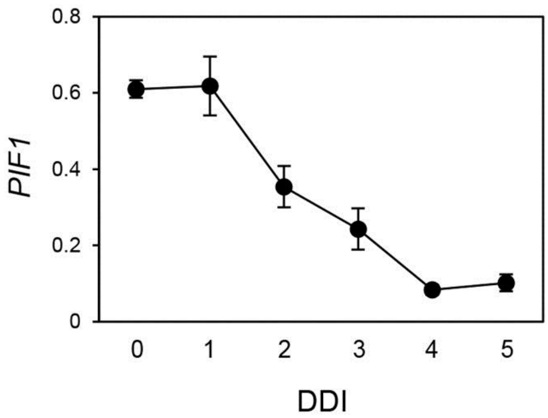

Supplemental Figure 8. The expression of Arabidopsis *PIF1* during DIS. Three-week-old WT (Col-0) whole plants grown under LD conditions were transferred to complete darkness, and 4th or 5th rosette leaves were sampled at 0DDI to 5 DDI for RT-qPCR. RT-qPCR analysis was used to measure the relative transcript levels of Arabidopsis *PIF1*, and transcript levels were normalized to the transcript levels of *GAPDH*. Mean and SD values were obtained from more than three biological replicates. DDI, day(s) of dark incubation.

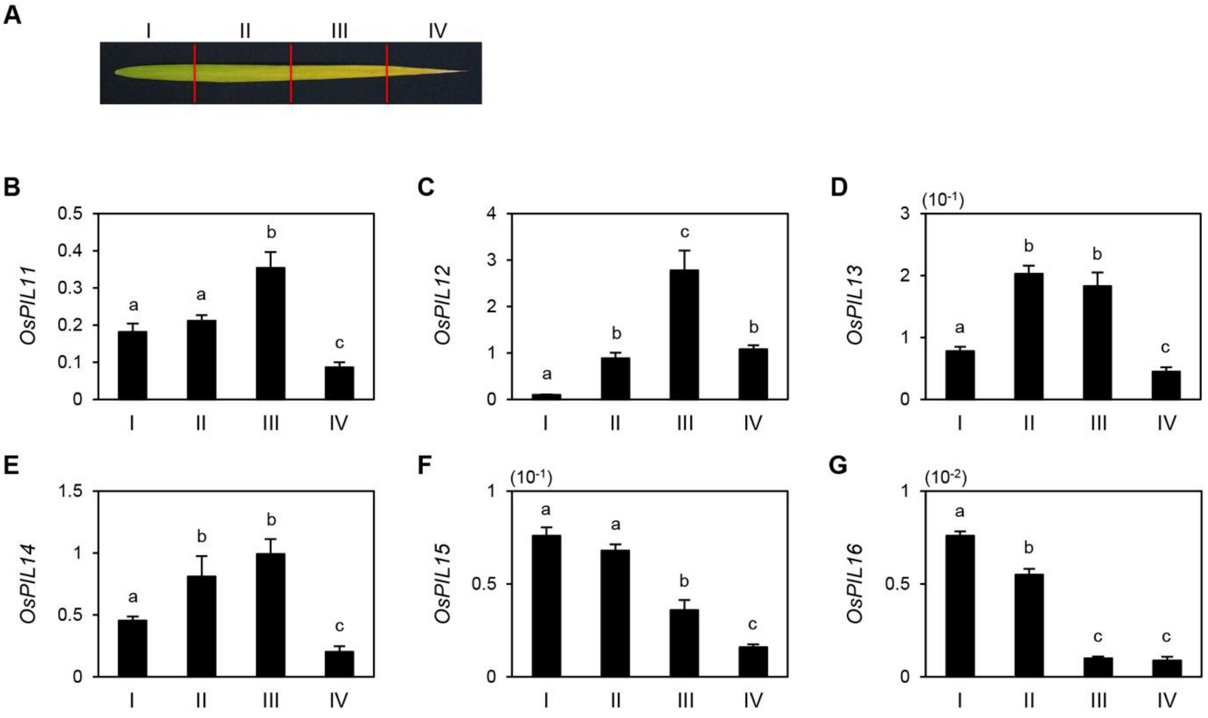

Supplemental Figure 9. Expression of six *OsPIF* genes in senescent rice leaves. (**A**) The senescent 2nd leaves of 90-DAG WT (cv. Dongjin) plants were sampled and divided into four parts for RT-qPCR (indicated as I, II, III, and IV). RT-qPCR analysis was used to measure the relative transcript levels of *OsPIL11* (**B**); *OsPIL12* (**C**); *OsPIL13* (**D**); *OsPIL14* (**E**); *OsPIL15* (**F**); and *OsPIL16* (**G**); and the transcript levels were normalized to the transcript levels of *OsUBQ5*. Mean and SD values were obtained from more than three biological replicates. This experiment was repeated twice with similar results. DAG, days after germination. The same letter in each bar graph indicates that means are not significantly different at the 0.05 level for Duncan's Multiple Range Test.

Supplemental Table 1. Primers used in this study.

	Forward Primer (5′->3′)	Reverse Primer (5′->3′)
OsABI5	CGAAGCTGAACTGAACTATC	CTGGCTGCCACCCCTATTTG
OsATG5	TCCTCAGGCACCAGATTCAG	GCCTTGCACCCTTACTAGCT
OsATG7	TCCAACCGAACACTGGATCA	GCTTGTGCCAGCAATCTCT
OsATG8a1	GTTGTTCGGAAGCGGATCAA	AGGTCATGTAGAGGAAGCCG
OsATG12	CGAGCAGAAGAAAGTCGTGG	ATCCTGGTGAATTTGTCGGC
OsEEL	GCAGAAGCGCATGATCAAGA	GACGCAGCTAGGGAATGTTG
OsEIN3	ATCTTCCCGGCAACCTACAA	CATGATCGTGGCATTGTCGT
OsLhcb1	CCATGTTCTCCATGTTCGGCTTCT	TAGGCCCAGGCGTTGTTGTTGA
OsLhcb4	TACCTGCAGTTCGAGCTGGAC	AGGCCGAACACCTCGGTGTA
OsNAP	AACCATTTCATCGCGAACAAC	CAGTGACGATCCCTGCAAGG
OsNYC1	GAATCCGTAATTGGGCTGAA	CTGGAAGAGGTCCACCTGAG
OsORE1	GTATTGCAACAAGGGCGAGA	GTGATGATCCATGCGTTGCT
OsPAO	GTGTTGCCTTCCACTGTCCT	ACTGAACATCCGCAGGAATC
OsPhyB	ATGGAACAGACACAATGCTT	AGCATACACCATATCAGCTT
OsPhyA	CCAAGAACAGCATTGGGGAAAACC	TTATTTTGCTGGAGCAGAAGCAAG
OsPIL11	GAGCTGTTCGGCGAAATGA	GTCACCGAACTCCGCCTT
OsPIL12	AAGGAAAGACTTGCAAGGGC	TCATCGTCTGCTGCCTCG
OsPIL13	AGGATGCTGAATGCGAGG	TTCTTGCAATGCGCGC
OsPIL14	ACGACGACGACGACATCG	GCTTGAACAGCGGGTAGC
OsPIL15	GACATCGTCGGTGTGCTC	TTCATCGTCAAGATCATCGTC
OsPIL16	GAGGGACAGCCACAACAAC	GCTCTGACAGGTTGTGGACTT
OsSAG12	GACCGGCACTTCCAGTTCTA	CCCCACGAGTTCTTCATCAG
OsSGR	AGGGGTGGTACAACAAGCTG	GCTCCTTGCGGAAGATGTAG
OsSGRL	CTCCAGCTCCAACGCTAAGTA	CTTCTTGTGCCTTTGGTGATT
OsUBQ5	ACCACTTCGACCGCCACTACT	ACGCCTAAGCCTGCTGGTT
PIF1	GGCGATGGGTATGGGTATGA	GACGGGTCAGAAGCATGAAC
GAPDH	TTGGTGACAACAGGTCAAGCA	AAACTTGTCGCTCAATGCAATC
